# Spirometry at diagnosis and overall survival in non‐small cell lung cancer patients

**DOI:** 10.1002/cam4.4808

**Published:** 2022-05-12

**Authors:** Ting Zhai, Yi Li, Robert Brown, Michael Lanuti, Justin F. Gainor, David C. Christiani

**Affiliations:** ^1^ Department of Environmental Health Harvard T.H. Chan School of Public Health Boston Massachusetts USA; ^2^ Department of Biostatistics University of Michigan School of Public Health Ann Arbor Michigan USA; ^3^ Pulmonary and Critical Care Division, Department of Medicine Massachusetts General Hospital and Harvard Medical School Boston Massachusetts USA; ^4^ Division of Thoracic Surgery, Department of Surgery Massachusetts General Hospital and Harvard Medical School Boston Massachusetts USA; ^5^ Massachusetts General Hospital Cancer Center and Department of Hematology & Oncology Massachusetts General Hospital and Harvard Medical School Boston Massachusetts USA

**Keywords:** epidemiology, lung cancer, prognostic factor, survival

## Abstract

Pulmonary function can predict all‐cause mortality, and chronic obstructive pulmonary disease (COPD) is associated with worse overall survival (OS) in non‐small cell lung cancer (NSCLC) patients. Though pre‐operative lung function is predictive of in‐hospital mortality following lung cancer surgery, its predictive utility for long‐term survival is unclear. The prognostic role of commonly used spirometry tests in survival of lung cancer also remains uncertain. This study evaluates the role of spirometry at lung cancer diagnosis in predicting OS of NSCLC patients. This was a retrospective study using data from the Boston Lung Cancer Study on newly diagnosed NSCLC patients with spirometry tests performed before cancer therapy (*n* = 2805). Spirometric test values, after being categorized using quartiles, were analyzed for association with OS using univariate and risk‐adjusted multiple regression models. Further, we analyzed OS by the status of COPD determined by spirometry, and, among those with COPD, by its stage defined by the Global Initiative for Chronic Obstructive Lung Disease criteria. Both univariate and multiple regression models demonstrated that lower quartiles of actual and percent predicted forced expiratory volume in 1 second and forced vital capacity at lung cancer diagnosis were significantly associated with worse OS. Spirometry‐determined COPD, and more advanced stage of COPD at lung cancer diagnosis were associated with worse lung cancer OS. The findings provide evidence that a good pulmonary function at diagnosis may help improve OS in NSCLC patients.

## Graphical abstract

Lung function has been associated with in‐hospital mortality following lung cancer surgery but the predictive role of lung function in long‐term survival of NSCLC patients remains unclear. Our analyses, based on a large lung cancer cohort, revealed that NSCLC patients with lower spirometry function at diagnosis were associated with worse overall survival. The funding provides evidence for evaluating lung function before the initiation of lung cancer therapy in the long‐term risk stratification of NSCLC patients.

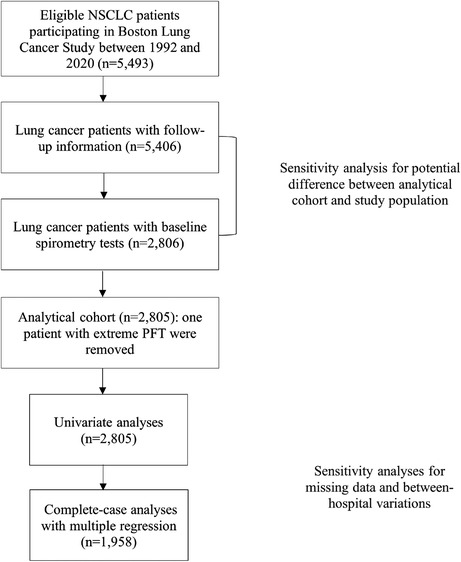

## 1 INTRODUCTION

Lung cancer is the leading cause of cancer‐related deaths, with a poor 5‐year survival rate of 18.8% in the United States in 2017.^1^ Non‐small cell lung cancer (NSCLC) accounts for 84% of diagnosed lung cancer and, in general, grows and spreads less aggressively than small cell lung cancer (SCLC).^1^ Surgical resection is a preferred therapy for stage I and II NSCLC patients, while systemic therapy with or without radiation therapy are mainly prescribed to patients in stage III and IV, respectively.^2^ In an effort to improve the survival rate, numerous studies have investigated the prognostic factors for survival of lung cancer, including age, sex, disease stage, and smoking.^3^ Identification of additional risk factors can help further control the mortality rate and better inform interventions to prolong survival in lung cancer patients.

Pulmonary function, as an important risk factor for overall mortality, has been widely utilized for lung disease diagnosis and management.^4^ Forced expiratory volume in 1 second (FEV_1_) and forced vital capacity (FVC) evaluate airway function and lung size, and are the two most commonly conducted pulmonary function tests (PFTs) in clinics. Preoperative spirometry has been shown to predict postoperative mortality.^5^ However, the association of pulmonary function with long‐term survival in lung cancer patients is less understood. Most of the available studies reached inconclusive association results, because these studies were typically small‐scale, targeted only early‐stage NSCLC patients, and used various PFT variables.^6^, ^7^, ^8^, ^9^, ^10^, ^11^, ^12^ Moreover, lung cancer patients, even with similar pulmonary function, may have different survival outcomes, suggesting that other prognostic factors play a role in survival of lung cancer and, therefore, should be accounted for. A larger‐scale study, with various stages of NSCLC patients, is needed to delineate the role of the commonly and easily used pulmonary function tests (i.e., spirometry) in predicting prognosis and potentially informing treatment plans.

Another potential prognostic factor of survival in lung cancer patients is chronic obstructive pulmonary disease (COPD), which is characterized by irreversible airflow obstruction. Our previous work reported that physician‐diagnosed COPD was associated with worse survival in early‐stage NSCLC patients receiving curative surgery.^13^ COPD is commonly diagnosed by using the Global Initiative for Chronic Obstructive Lung Disease (GOLD) criteria,^14^ which utilize FEV_1_ and FVC to assess pulmonary function. It is unclear whether more advanced GOLD stages are associated with worse overall survival (OS) and whether the association remains the same for advanced stage NSCLC patients or those receiving chemotherapy or radiation therapy.

This study examined the prognostic role of spirometry at lung cancer diagnosis in the long‐term OS of NSCLC patients, with a hypothesis that poor pulmonary function assessed by spirometry tests is linearly associated with worse OS. We further tested the association between COPD and OS among NSCLC patients with different stages and treatments and across various GOLD stages of COPD.

## 2 METHODS

### 2.1 Participants

**FIGURE 1:**
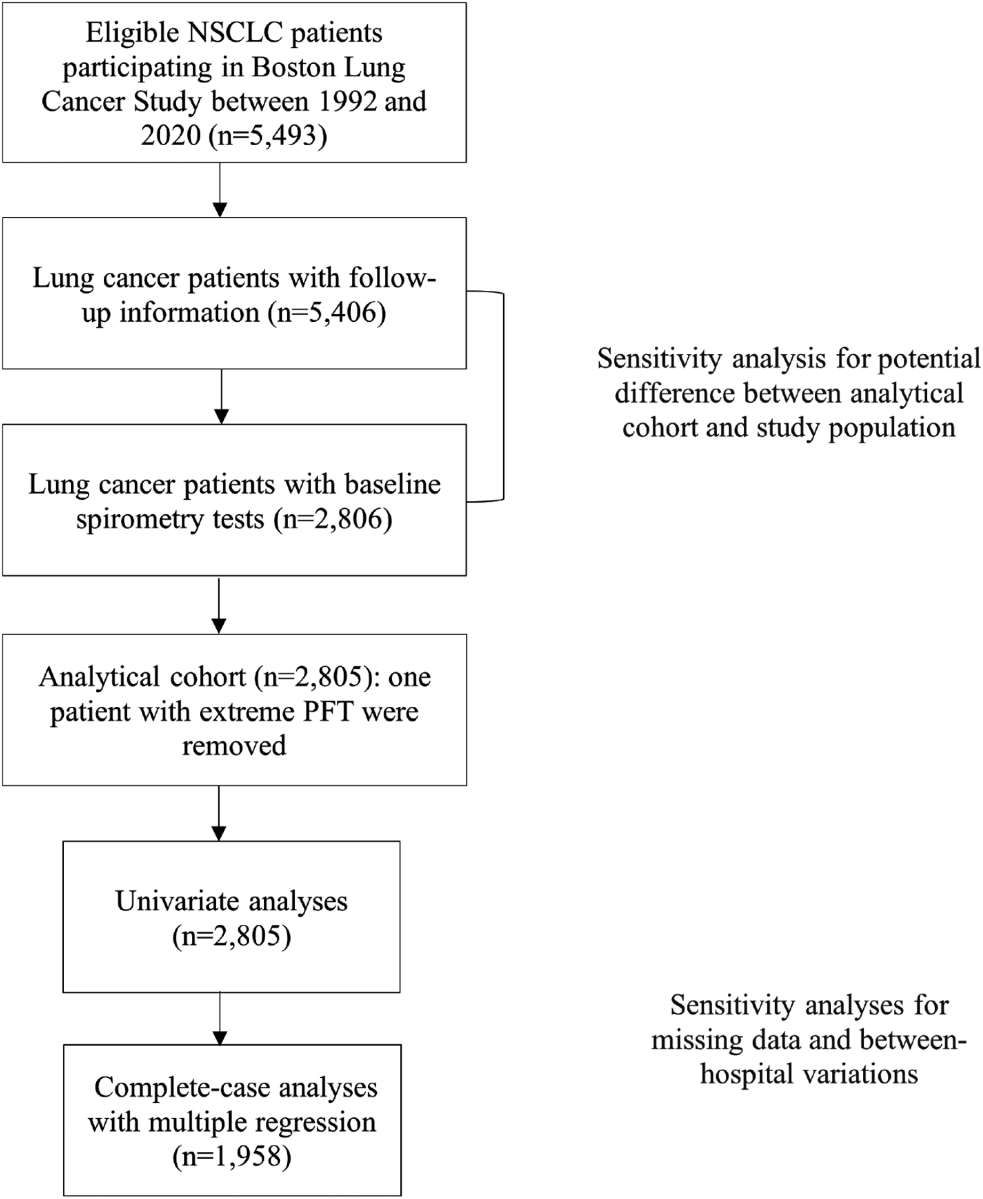
Inclusion for the study population and analytical cohort

Included in this study were newly diagnosed lung cancer patients, who were recruited into a cancer epidemiology cohort (Boston Lung Cancer Study, BLCS) at Massachusetts General Hospital (MGH) starting in 1992. All patients were at least 18 years old at enrollment and > 95% of them were White. Demographic information, for example, age, sex, body mass index (BMI), smoking status, and intensity, was collected at enrollment by trained staff using a standardized questionnaire. Clinical information was retrieved retrospectively from medical records, including tumor histology, clinical stage, and types of initial treatment for lung cancer. Informed consent for the collection of follow‐up data from patients or their surrogates was obtained. Figure 1 details how the analytical cohort was obtained: among 5493 patients with histologically confirmed NSCLC, 5406 patients had available follow‐up information by July 2020, and we restricted our analysis to those who had at least one spirometry test performed before initiating lung cancer treatment (*n* = 2805).

### 2.2 PFTs and survival data collection

Spirometry data were collected retrospectively from PFT reports or medical records of each lung cancer patient, including actual FEV_1_ and percent predicted FEV_1_%, actual FVC and percent predicted FVC %, as well as post‐bronchodilator (post‐bd) FEV_1_, FEV_1_%, FVC, and FVC %. We restricted the spirometry tests to those performed before initial lung cancer treatment, as the results of PFTs performed after treatment may be confounded by the treatment. The test rate for post‐bd PFTs was low among patients in the analytic cohort (Table 2). One patient with an extreme FVC value of 16 L, likely due to an error, was removed from the analytical cohort.

In our analysis, obstructive lung disease, heretofore termed generically as “COPD” was determined by spirometry tests and was further staged by the GOLD criteria.^14^ Specifically, a COPD pattern was determined if the ratio of FEV_1_/FVC < 70%, and COPD was staged to be 1, 2, 3, and 4 if percent predicted FEV_1_% ≥ 80%, 50% ~ 79%, 30% ~ 49%, and < 30%, respectively.

OS was defined to be the time lag between the date of pathological diagnosis of lung cancer and the date of death (event) or the last known date alive (censor), whichever came first. Data were collected from the following sources as previously described^15^: MGH patient and outpatient records, MGH tumor registry, social security death index, primary physician's office, and patient or family contact.

### 2.3 Statistical analysis

Data were compared between patients with and without spirometry‐determined COPD by using *t*‐tests for continuous variables and chi‐squared tests for categorical variables. Cox proportional hazard models were used for identifying the associations of spirometry tests and demographic and clinical characteristics with OS. The associations were assessed by using univariate and multiple Cox regression models. To reduce the impact of outliers, values of each spirometry test were categorized into four groups using quartiles as the cutoffs, and the fourth quartile group (75–100%) was used as the reference group in the models. Variables included in the multiple Cox models were those with significant associations with OS identified by univariate models or believed to confound the association between pulmonary function and OS based on prior knowledge. They were age, sex, BMI, smoking status (previous smoker, or current smoker vs. never smoker), pack‐years, NSCLC histological subtypes (bronchioloalveolar carcinoma, squamous cell, large cell, NSCLC‐unspecified vs. adenocarcinoma), clinical stages (II, III, or IV vs I), and lung cancer treatments (surgery + chemo/radiation, or chemo/radiation vs. surgery only). The goodness‐of‐fit for the Cox models was evaluated by the plots of deviance residuals. To avoid collinearity, each spirometry test was separately included in a multiple regression model without including the other spirometry tests.

The proportional hazard (PH) assumption was further tested for each variable by checking the significance of its interaction with time. Variables that violated the PH assumption were either modeled with time interaction terms (i.e., age, stage, and lung cancer treatment) or stratified upon (i.e., NSCLC histological subtypes). The NSCLC histological subtypes were stratified on rather than adjusted for as a covariate in the Cox models, because their effects, viewed as confounding effects, were not the main focus of this study, and stratification can eliminate these confounding effects without explicitly modeling them.^16^ The association between spirometry‐determined COPD and OS was tested by using the log‐rank test and was presented by using Kaplan–Meier curves.

With no evidence for informative missingness, we conducted complete‐case analyses as our main analyses, given a large sample size with a complete‐case rate of 70%. We further performed imputation for the missing values as sensitivity analyses and compared them with the complete‐case analysis results; see details in the next section of Sensitivity Analyses.

All *p*‐values were two‐sided and *p* < 0.05 was considered statistically significant. Statistical analyses were conducted using SAS (version 9.4; SAS Institute Inc.) and R (version 4.0.2; R Foundation for Statistical Computing).

### 2.4 Sensitivity analyses

To validate the associations between the spirometry tests and OS obtained from the main analyses, we refit the univariate and multiple regression models by using post‐bd spirometry tests. We imputed missing covariates based on the mean, median, or mode of the observed data^17^ and compared the imputation‐based results with the complete‐case results. The predictive performance of Cox models with predictors at the baseline was compared by using Harrell's C‐index.^18^ To investigate whether there was potential selection bias with our analytic cohort, we compared the demographic and clinical characteristics and survival between NSCLC patients with available spirometry tests in our analytical cohort and NSCLC patients from the entire cohort without spirometry tests. Accounting for the potential heterogeneity in spirometry tests between PFTs conducted within and outside MGH and based on the sources of PFTs, we created an index to label in‐hospital and out‐hospital spirometry. We further compared the demographic and clinical characteristics between patients with in‐ and out‐hospital PFTs, and tested the effect of between‐hospital variations by examining the interaction between this index and each spirometry variable.

## 3 RESULTS

### 3.1 Patient characteristics

Demographic and clinical information for the 2805 NSCLC patients in the analytic cohort is shown in Table 1, with their spirometry results at diagnosis summarized in Table 2. At diagnosis, the mean (SD) age of the analytical cohort was 67.40 (10.02) years, with an average of BMI 26.64 (5.39) kg/m^2^, 49.73% of the patients were male, 59.80% of the patients were former smokers, 28.58% were current smokers, and adenocarcinoma (59.61%) was the most prevalent histological subtype, followed by squamous cell cancer (22.35%). Most of the patients were diagnosed at stage I (55.75%), and 61.74% of the patients only received surgical treatment for lung cancer.

**TABLE 1:** Characteristics of NSCLC patients in the analytic cohort

Variable	ALL patients (*n* = 2805)	Patients with COPD (*n* = 1275)	Patients without COPD (*n* = 1530)	*p*
Age	67.40 (10.02)	68.65 (9.41)	66.36 (10.38)	<0.0001
Male sex	1359 (49.73)	646 (52.22)	713 (47.66)	0.0176
BMI	26.64 (5.39)	25.74 (5.17)	27.37 (5.46)	<0.0001
Smoking status				<0.0001
Never smoker	313 (11.62)	66 (5.38)	247 (16.83)	
Previous smoker	1611 (59.80)	735 (59.95)	876 (59.67)	
Current smoker	770 (28.58)	425 (34.67)	345 (23.50)	
Pack‐years	37.42 (15.67–58.50)	47.00 (30.00–69.17)	29.00 (5.00–49.00)	<0.0001
NSCLC histology				<0.0001
Adenocarcinoma	1672 (59.61)	685 (53.73)	987 (64.51)	
BAC	194 (6.92)	72 (5.65)	122 (7.97)	
Squamous cell	627 (22.35)	357 (28.00)	270 (17.65)	
Large cell	112 (3.99)	64 (5.02)	48 (3.14)	
NSCLC‐unspecified	200 (7.13)	97 (7.61)	103 (6.73)	
Stage				0.7652
I	1547 (55.75)	692 (54.88)	855 (56.47)	
II	539 (19.42)	246 (19.51)	293 (19.35)	
III	536 (19.32)	254 (20.14)	282 (18.63)	
IV	153 (5.51)	69 (5.47)	84 (5.55)	
Lung cancer treatment				0.0023
Surgery only	1657 (61.74)	721 (59.64)	935 (63.43)	
Surgery + Chemo/Radiation	491 (18.29)	256 (21.17)	235 (15.94)	
Chemo/Radiation	536 (19.97)	232 (19.19)	304 (20.62)	

Abbreviations: BAC, bronchioloalveolar carcinoma; BMI, body mass index; COPD, chronic obstructive pulmonary disease; NSCLC, non‐small cell lung cancer.

Data are presented as mean (standard deviation) for age, BMI, and median (interquartile range) for pack‐years, No. (%) for categorical variables. They were summary statistics of the observed data (with missing rate ranging from 1.07% to 19.64%, respectively).

**TABLE 2:** Spirometry distribution in the analytical cohort

			Cutoff values in each quartile			
Spirometry	N	Mean (SD)	0–25%	25–50%	50–75%	75–100%
FEV_1_	2805	2.11 (0.75)	0.37–1.57	1.58–2.02	2.03–2.59	2.60–5.45
FEV_1_%	2805	78.29 (23.25)	4–62	63–79	80–93	94–163
FVC	2805	3.03 (0.94)	0.66–2.36	2.37–2.93	2.94–3.61	3.62–8.20
FVC %	2805	86.41 (19.99)	2–73	74–86	87–99	100–171
FEV_1_ post‐bd	906	1.94 (0.68)	0.48–1.42	1.43–1.89	1.90–2.36	2.37–4.31
FEV_1_% post‐bd	900	71.90 (21.90)	22–57	58–71	72–86	87–154
FVC post‐bd	905	2.97 (0.91)	0.99–2.32	2.33–2.88	2.89–3.60	3.61–6.67
FVC % post‐bd	899	83.49 (19.38)	32–70	71–83	84–95	96–157

Abbreviations: FEV_1_, forced expiratory volume in 1 second; FEV_1_%, percent predicted FEV_1_; FVC %, percent predicted FVC; FVC, forced vital capacity; post‐bd, post bronchodilator; SD, standard deviation.

During the follow‐up, a total of 1665 deaths were observed with a median survival of 64.3 months (95% confidence interval [CI]: 59.45–70.07 months), and a 1‐, 5‐, and 10‐year survival rate of 85.6%, 51.8%, and 33.8%, respectively. The median follow‐up length was 40.7 months (range, 0.07–281.54 months). Age, sex, BMI, smoking status, NSCLC histological subtypes, clinical stages, and post‐diagnosis treatments were all significantly associated with OS as detected by univariate analyses (Table 3).

**TABLE 3:** Univariate Cox regression analysis exploring prognostic factors of OS in lung cancer

Variable	Hazard ratio	95%CI	*p* value
Age	1.025	1.019, 1.030	<0.0001
Male sex	1.270	1.152, 1.400	<0.0001
BMI	0.984	0.974, 0.993	0.0011
Smoking status			
Never smoker	–	–	–
Previous smoker	1.579	1.320, 1.890	<0.0001
Current smoker	1.878	1.557, 2.264	<0.0001
Pack‐years	1.007	1.005, 1.008	<0.0001
NSCLC histology			
Adenocarcinoma	–	–	–
BAC	0.758	0.631, 0.911	0.0031
Squamous cell	1.628	1.454, 1.823	<0.0001
Large cell	1.743	1.412, 2.151	<0.0001
NSCLC‐unspecified	1.224	1.013, 1.479	0.0364
Stage			
I	–	–	–
II	2.464	2.179, 2.787	<0.0001
III	2.417	2.138, 2.734	<0.0001
IV	4.592	3.795, 5.557	<0.0001
Lung cancer treatments			
Surgery only	–	–	–
Surgery + Chemo/Radiation	3.974	3.524, 4.481	<0.0001
Chemo/Radiation	1.298	1.143, 1.473	<0.0001

Abbreviations: BAC, bronchioloalveolar carcinoma; BMI, body mass index; CI, confidence interval; NSCLC, non‐small cell lung cancer.

For each categorical variable, the first level listed in the table was used as the reference group.

### 3.2 PFTs and lung cancer survival

**FIGURE 2:**
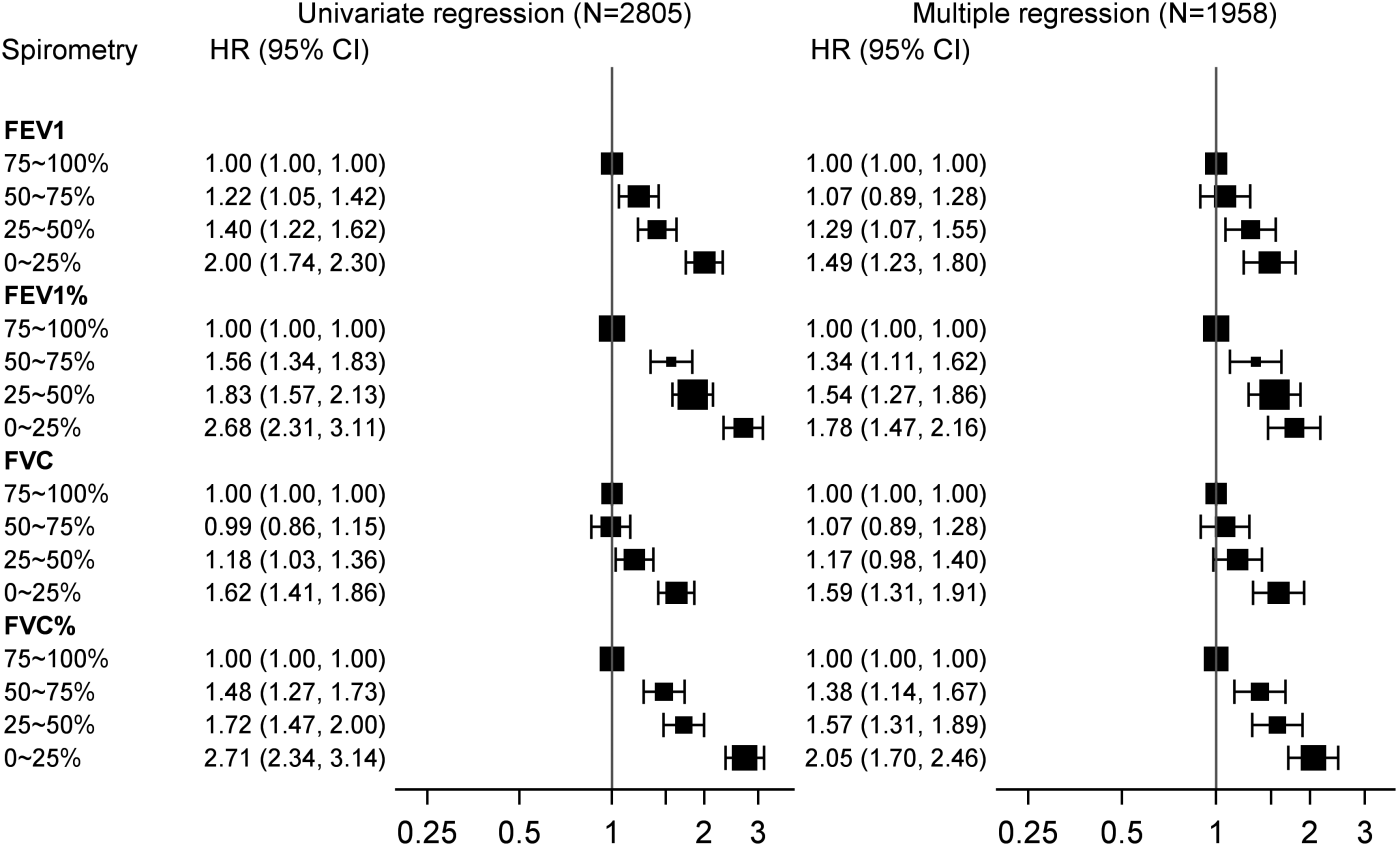
HRs of OS for each spirometry test in univariate (*N* = 2805) and stratified multiple regression models (complete‐case analyses, *N* = 1958). Variables adjusted in the multiple regression models are age, sex, BMI, smoking status (previous smoker, or current smoker vs. never smoker), pack‐years, cancer stage (II, III, or IV vs. I), treatments (surgery + chemo/radiation, or chemo/radiation vs. surgery only), and time interaction terms with age, stage, and treatment; the multiple regression model was further stratified on NSCLC histological subtypes. Abbreviations: HR: hazard ratio; CI: confidence interval; FEV_1_: forced expiratory volume in 1 second; FEV_1_%: percent predicted FEV_1_; FVC: forced vital capacity; FVC %: percent predicted FVC

Figure 2 shows the crude and adjusted HRs of each pre‐bd spirometry test based on our analytical cohort. In the univariate models, compared with the highest quartile group (75–100%), lower quartiles of PFTs (i.e., FEV_1_, FEV_1_%, FVC, and FVC%) were associated with higher mortality. Our multiple regression models showed that these associations remained true, after adjusting for age, sex, BMI, smoking status, clinical stages and lung cancer treatments and stratified on NSCLC histological subtypes. Deviance residuals suggested adequate goodness‐of‐fit of these multiple regression models (Figure E3).

The post‐bd spirometry showed a “dose‐dependent” association (i.e., larger value, better survival, and vice‐versa) with OS in both univariate and multiple regression models (Figure E1); and the Harrell's C‐index for multiple regression models was almost identical to those in the main results (Table E1). The imputation‐based results (Figure E2) agreed with those of the complete‐case analyses, and the models with imputed values had slightly lower C‐statistics (Table E1). This was expected as imputed values were prone to measurement errors and might reduce the predictiveness of models.^19^

Compared with NSCLC patients without valid spirometry tests in the BLCS cohort, patients in our analytical cohort were older, had higher BMI, more likely to be smokers, more likely to have NSCLC of bronchioloalveolar carcinoma or squamous cell type, and more likely to be in earlier stages and receive surgical treatment (Table E2). As a result, OS was lower in patients without valid spirometry tests in the BLCS cohort (median survival [95%CI]: 28.8 [26.7, 31.7] vs. 64.3 [59.5, 70.1] months), and the difference remained significant after adjusting for covariates used in the multiple regression analyses (HR comparing patients with and without valid spirometry tests [95%CI] = 0.748 [0.691, 0.810]).

With regard to the differences between patients with PFT performed within MGH and outside MGH, the former tended to be older, heavy smokers, less likely to have adenocarcinoma, and more likely to receive conjunctive therapies (Table E2). The source of PFT records was not associated with OS in the multiple regression models as its interaction with each spirometry test was not statistically significant (results not shown).

### 3.3 Spirometry‐determined COPD and lung cancer survival

Compared with patients without spirometry‐defined COPD at lung cancer diagnosis (Table 1), patients with COPD were older (mean ± SD of age in COPD vs. non‐COPD: 68.65 ± 9.41 vs. 66.36 ± 10.38 years), more likely to be male (COPD vs. non‐COPD: 52.22% vs. 47.66%), leaner (mean ± SD of BMI in COPD vs. non‐COPD: 25.74 ± 5.17 vs. 27.37 ± 5.46), and more likely to be current smokers (COPD vs. non‐COPD: 34.67% vs. 23.50%). Patients with COPD had a higher proportion of squamous cell cancer (COPD vs. non‐COPD: 28.00% vs. 17.65%), a lower proportion of adenocarcinoma types (COPD vs. non‐COPD: 53.73% vs. 64.51%), and were more likely to receive surgery plus chemo/radiation therapies (COPD vs. non‐COPD: 21.17% vs. 15.94%). There were no significant differences between patients with and without COPD in tumor stage.

**FIGURE 3:**
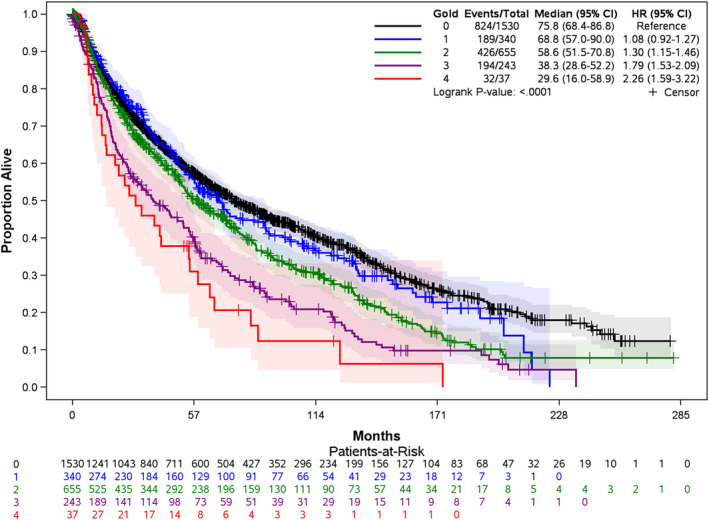
Kaplan–Meier curve of OS by COPD stage (*N* = 2805). Advanced stages of COPD were associated with significantly shorter OS in lung cancer patients. Abbreviations: GOLD: Global Initiative for Chronic Obstructive Lung Disease

The median survival was 75.8 months (95% CI: 68.4–86.8 months) for patients without COPD, whereas, among those with COPD, from their GOLD stages 1 to 4, the median survival was 68.8 months (95% CI: 57.0–90.0 months), 58.6 months (95% CI: 51.5–70.8 months), 38.3 months (95% CI: 28.6–52.2 months), and 29.6 months (95% CI: 16.0–58.9 months), respectively. More advanced COPD stages were associated with poorer survival (Figure 3).

## 4 DISCUSSION

We found that FEV_1_, FEV_1_%, FVC, and FVC % were associated with OS among NSCLC patients in a quantitative, dose‐dependent manner, even after adjusting for the other well‐studied prognostic factors for lung cancer and that more advanced stages of spirometry‐determined COPD were associated with worse OS. Specifically, lower quartiles of FEV_1_, FEV_1_%, and FVC, FVC % were associated with worse OS. Similar results have been found for both lung cancer patients^13^ and for the general population.^20^ Moreover, Mannino *et el*. reported that both pre‐ and post‐bronchodilator spirometry predicted overall survival in the Lung Health Study.^21^

Prior studies on associations between spirometry and survival in NSCLC produced inconclusive results, likely because the majority of them were conducted on small populations. For example, Berry et al.^7^ and Ferguson et al.^8^ reported non‐associations between FEV_1_% and OS in NSCLC patients after surgery, but Iizasa et al.^22^ found FEV1% is the most significant prognostic factor in stage I NSCLC; Warner et al.^9^ reported FEV1% as a dominant predictor of 180‐day survival in NSCLC patients after concurrent chemoradiation therapy, but Ackerson et al.^6^ did not find a significant association between FEV1% and OS in NSCLC with either radiation therapy or surgery. Among studies that examined impaired pulmonary function defined by FEV_1_%, most of them reported significant associations between impaired pulmonary function and survival in lung cancer patients,^23^, ^24^, ^25^, ^26^, ^27^, ^28^, ^29^ but the cutoff value of FEV_1_% varied across these studies. FVC% was less studied, but Guo et al.^11^ and Gao et al.^10^ showed FVC% < 80% was a significant negative prognostic factor of OS in NSCLC patients after surgical resections. Same situations also applied to the inconclusive findings about the relationship between COPD and survival in lung cancer patients. For patients received surgical resection, similar to previously reported in BLCS,^13^ both Bugge et al.^30^ and Wang et al.^31^ found decreased survival in patients with severe COPD, and study from López‐Encuentra et al.^32^ also suggested COPD as a prognostic factor in a larger cohort of 2994 lung cancer patients. Shorter overall survival with COPD was also found in Yi et al.^33^ study among advanced NSCLC patients. However, no impact of coexisting COPD has been reported in a few studies,^12^, ^34^, ^35^, ^36^ and Shine et al.^37^ reported in NSCLC treated with pembrolizumab, coexisting with COPD was associated with a significantly longer survival. In addition to limited sample size, cancer stage or treatment might also explain the controversy, as most inconclusive results were generated in advanced stage patients receiving treatments other than surgery.

The raw values of FEV_1_ and FVC did not retain dose–response associations with OS because of the impacts of outliers, but the lowest quartiles of FEV_1_ and FVC were significantly associated with lower OS, suggesting the usefulness of categorizing continuous variables in the presence of outlier values. In our main analyses, we used the spirometry tests done before bronchodilator as pre‐bronchodilator spirometry is the most widely reported. We explored the role of spirometry after bronchodilator in 43.5% of the analytical cohort with valid post‐bd spirometry tests, and observed similar dose–response relationships as pre‐bd spirometry tests (Figure E1), with similar predictive performances (Table E1).

We implemented QC for data retrieving, but the measurement errors may still exist, especially among patients with PFTs conducted outside MGH. We conducted a validation study by assessing the impact of potential between‐hospital variations in PFTs among a sample of 1158 patients with verified sources of PFTs, and found that neither the PFT source indicator nor the interaction between the source indicator and spirometry test was significantly associated with OS. Our large size cohort empowered us to conduct complete‐case analyses, and the obtained models were slightly more predictive than those based on data imputed by mean or mode (Figure E2; Table E1) or by multiple imputation based on the observed covariates (data not shown).

There are some limitations with the study. First, because of some unmeasured prognostic factors (e.g., performance status and physical activity), the estimated associations between pretreatment pulmonary function and long‐term survival in NSCLC patients could be biased. In our analysis, we adjusted for confounders to the extent possible. In addition to demographic variables, we also adjusted for cancer stage, NSCLC histology, initial treatments and surgery procedures, and controlled for their potential time‐varying effects on survival. As the treatment options could be influenced by patients' pulmonary function, which makes treatment a mediator for the spirometry and survival relationship, by controlling for treatment, we estimated a “direct effect” of spirometry on survival than a “total effect” (which could be decomposed into direct effect of spirometry on survival and indirect effect mediated through treatment). And because of the observed significant associations, our results are still informative and meaningful. Second, there might be selection bias with the patients included in our analysis as subjects with advanced stages of lung cancer were less likely to receive PFTs from chemo/radiation therapies. Therefore, we are cautious about the generalizability of the results to a general lung cancer population. However, we did perform several sensitivity analyses and observed similar dose–response relationships between baseline pulmonary function and overall survival as compared to the main results. Moreover, with our main interest focused on pulmonary function, the current findings were still useful for verifying the important role of the baseline pulmonary function in lung cancer prognosis. Third, as specific causes of death were scarcely documented in this population, we focused on overall mortality instead of lung cancer‐specific death. Because of the aggressiveness of lung cancer especially among the late‐stage lung cancer patients, it is reasonable to assume that the majority of deaths in our study were likely due to lung cancer.

Compared to other related studies, one strength of this study was a large cohort with FEV_1_ (FEV_1_%) and FVC (FVC %), two most common PFTs in clinical practice. The independent impact of spirometry on OS is valuable for evaluating the long‐term prognosis of lung cancer. Previous studies, which either used continuous or a single cut‐off of PFT or failed to account for various histological types and treatment options for lung cancer, might have introduced residual confounding. In contrast, we retrieved more complete spirometry results from a long‐established lung cancer cohort with complete information on follow‐up, and were able to explore a comprehensive set of prognostic factors. The similar HRs of each spirometry obtained for patients receiving various treatments further indicates the possible generalizability to NSCLC patients with different cancer therapies post‐diagnosis. Last, using spirometry‐defined COPD, we confirmed our earlier study relating physician‐diagnosed COPD to lung cancer prognosis.^13^

Our analyses of a large lung cancer cohort revealed that in NSCLC patients lower pulmonary function at diagnosis was associated with worse survival, even after accounting for age, sex, smoking status, tumor histology, cancer stage, and types of treatment. Lower FEV_1_, FEV_1_%, and FVC and FVC %, were associated with worse OS, and are therefore potential biomarkers for lung cancer survival. Moreover, COPD, defined by spirometry, was significantly associated with OS. Based on the study finding, we recommend spirometry tests to be regularly conducted for risk stratification of lung cancer patients upon their diagnosis, regardless of their stage, which will further inform clinical decisions for appropriate treatment and care strategies to prolong their long‐term survival. And spirometry might be extremely helpful for patients in advanced stages since a more personalized treatment strategy for them could better improve their survival outcomes. As pulmonary function could be an early biomarker for disease and aging, further research could dive into the observed role of pulmonary function in developing biomarkers and treatment targets for lung cancer prognosis.

## CONFLICT OF INTEREST

The authors have no conflict of interest to declare.

## AUTHOR CONTRIBUTIONS

Conception and design: TZ, DC.

Collecting the data: TZ, RB, DC.

Conducting the statistical analyses: TZ.

Writing the draft manuscript and revision: TZ, YL, DC.

Interpretation of results and revision of the manuscript: all authors.

Final approval of the submitted version: all authors.

## ETHICS STATEMENT

The study was approved by the institutional review board of MGH (Partners Human Research Committee, Protocol No. 1999P004935/MGH).

## Supporting information


**Appendix S1** Supplementary Information

## Data Availability

The data that support the findings of this study are available upon reasonable request from the corresponding author, Dr. David Christiani (dchris@hsph.harvard.edu).
