# Supplementary material for: Spirometry at diagnosis and overall survival in non‐small cell lung cancer patients
Source: Cancer Med. 2022 May 12;11(24):4796–805. doi: 10.1002/cam4.4808 (PMC9761088; doi:10.1002/cam4.4808)
Supplement: Supplementary file 1 — Appendix S1 Supplementary Information [file CAM4-11-4796-s001.docx]

# ONLINE DATA SUPPLEMENT

| **Table E1.** **Harrell’s C-index for each model in the sensitivity analysis.** | | | |
| --- | --- | --- | --- |
| **Spirometry** | **Main results** | **Post-bd spirometry** | **Imputation** |
| FEV_1_ | 0.736 | 0.734 | 0.719 |
| FEV_1_% | 0.741 | 0.739 | 0.724 |
| FVC | 0.738 | 0.737 | 0.719 |
| FVC % | 0.744 | 0.741 | 0.727 |
| Abbreviations: FEV_1_ = forced expiratory volume in 1 second; FEV_1_% = percent predicted FEV_1_; FVC = forced vital capacity; FVC % = percent predicted FVC; post-bd=post bronchodilator. | | | |

| **Table E2.** **Comparison for demographic and clinical characteristics between NSCLC patients with /without valid spirometry test at diagnosis in the BLCS cohort (Spirometry/ non- Spirometry) and between NSCLC patients with /without in-hospital spirometry test in the analytical cohort (In-hospital/ Out-hospital).** | | | | |
| --- | --- | --- | --- | --- |
| **Variable** | **Spirometry**  **(*n* = 2,805)** | **non-Spirometry**  **(*n* = 2,600)** | **In-hospital**  **(*n* = 717)** | **Out-hospital**  **(n = 441)** |
| Age*^ | 67.40 (10.02) | 64.86 (10.98) | 67.73 (10.17) | 64.84 (10.89) |
| Male sex | 1,359 (49.73) | 1241 (49.03) | 325 (46.30) | 194 (47.32) |
| BMI* | 26.64 (5.39) | 26.10 (5.37) | 26.28 (5.35) | 26.57 (5.26) |
| Smoking status* | | | |  |
| Never smoker | 313 (11.62) | 419 (16.88) | 75 (10.82) | 59 (14.71) |
| Previous smoker | 1,611 (59.80) | 1,366 (55.04) | 354 (51.08) | 209 (52.12) |
| Current smoker | 770 (28.58) | 697 (28.08) | 264 (38.10) | 133 (33.17) |
| Pack-years*^ | 37.42 (15.67 - 58.50) | 32.00 (6.68 – 54.03) | 38.98 (19.24 – 59.78) | 30.46 (6.87-47.98) |
| NSCLC histology *^ | | | |  |
| Adenocarcinoma | 1,672 (59.61) | 1,642 (62.46) | 342 (47.70) | 270 (61.22) |
| BAC | 194 (6.92) | 114 (4.38) | 93 (12.97) | 19 (4.31) |
| Squamous cell | 627 (22.35) | 473 (18.19) | 174 (24.27) | 87 (19.73) |
| Large cell | 112 (3.99) | 102 (3.92) | 24 (3.35) | 12 (2.72) |
| NSCLC-unspecified | 200 (7.13) | 287 (11.04) | 84 (11.72) | 53 (12.02) |
| Stage* | | | |  |
| I | 1,547 (55.75) | 905 (35.27) | 332 (46.83) | 216 (49.54) |
| II | 539 (19.42) | 630 (24.55) | 156 (22.00) | 97 (22.25) |
| III | 536 (19.32) | 431 (16.80) | 164 (23.13) | 96 (22.02) |
| IV | 153 (5.51) | 600 (23.38) | 57 (8.04) | 27 (6.19) |
| Lung cancer treatments*^ |  |  |  |  |
| Surgery only | 1,657 (61.74) | 1,180 (45.38) | 339 (47.28) | 241 (54.65) |
| Surgery + Chemo/Radiation | 491 (18.29) | 1,098 (42.23) | 195 (27.20) | 82 (18.59) |
| Chemo/Radiation | 536 (19.97) | 322 (12.38) | 183 (25.52) | 118 (26.76) |
| Data are presented as mean (standard deviation) for age, BMI, and median (interquartile range) for pack-years, No. (%) for categorical variables. They were summary statistics of the observed data (with missing rate ranging from 1.07% to 19.64%, respectively).  *P<0.05 for the comparison between patients with valid spirometry tests and without in the BLCS cohort;  ^P<0.05 for the comparison between patients with spirometry performed within MGH and outside from MGH in the analytical cohort.  Abbreviations: BMI = body mass index; BAC = bronchioloalveolar carcinoma; NSCLC = non-small cell lung cancer. | | | | |

# Figure Legends

**Figure E1.** **HRs of OS for each post-bd spirometry test in univariate (N=906) and multiple regression models (N=596).** Variables adjusted in the multiple regression models are age, sex, BMI, smoking status, pack-years, stage, treatments, and time interaction terms with age, stage and treatment; the multiple regression model was further stratified on NSCLC histological subtypes.

Abbreviations: HR = hazard ratio; CI = confidence interval; FEV_1_ = forced expiratory volume in 1 second; FEV_1_% = percent predicted FEV_1_; FVC = forced vital capacity; FVC % = percent predicted FVC; post-bd=post bronchodilator.

**Figure E2.** **Comparison between HRs from complete-case analysis (N=1,958) and HRs from analysis after missing value imputation (simple imputation with mean/median/mode, N=2,805).** Variables adjusted in the multiple regression models are age, sex, BMI, smoking status, pack-years, stage, treatments, and time interaction terms with age, stage and treatment; the regression model was further stratified on NSCLC histological subtypes.

Abbreviations: HR = hazard ratio; CI = confidence interval; FEV_1_ = forced expiratory volume in 1 second; FEV_1_% = percent predicted FEV_1_; FVC = forced vital capacity; FVC % = percent predicted FVC.

**Figure E3.** **Deviance plots for Cox models in the main analyses.** FEV_1_ (A), FEV_1_% (B), FVC (C), or FVC % (D) was modeled with adjustments for age, sex, BMI, smoking status, pack-years, stage, and stratified on NSCLC histological types.

**
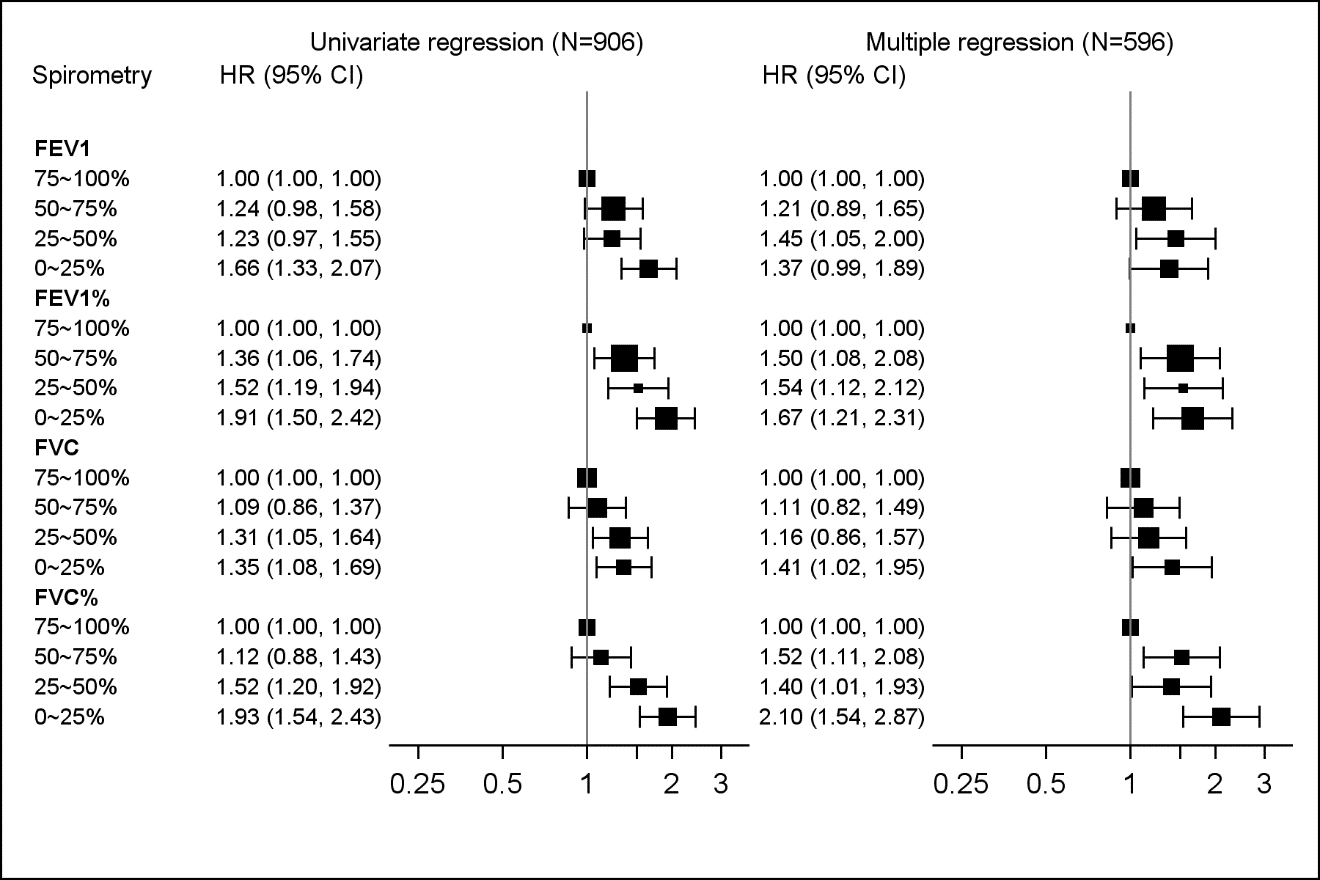
**

**Figure E1.** **HRs of OS for each post-bd spirometry test in univariate (N=906) and multiple regression models (N=596).**

**
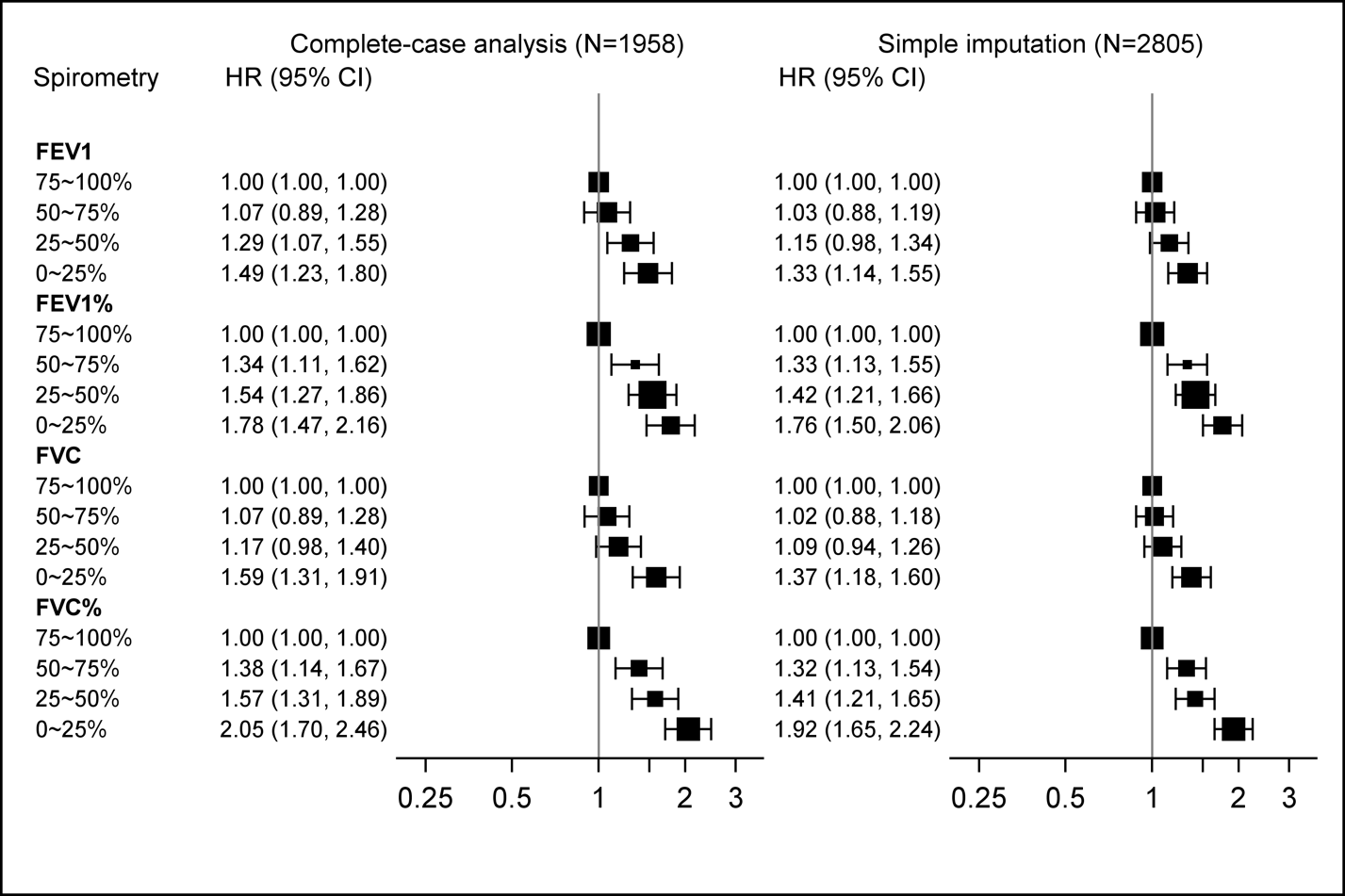
**

**Figure E2. Comparison between HRs from complete-case analysis (N=1,958) and HRs from analysis after missing value imputation (simple imputation with mean/median/mode, N=2,805).**

| A  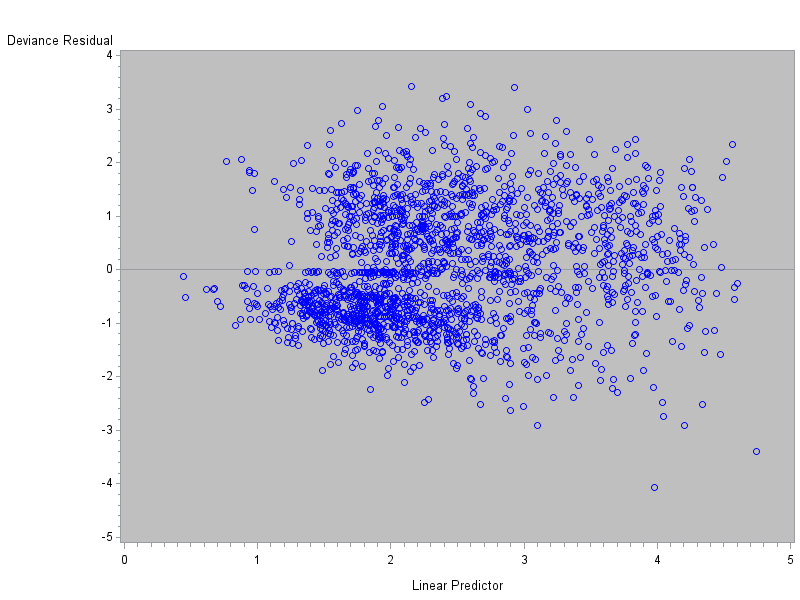 | B  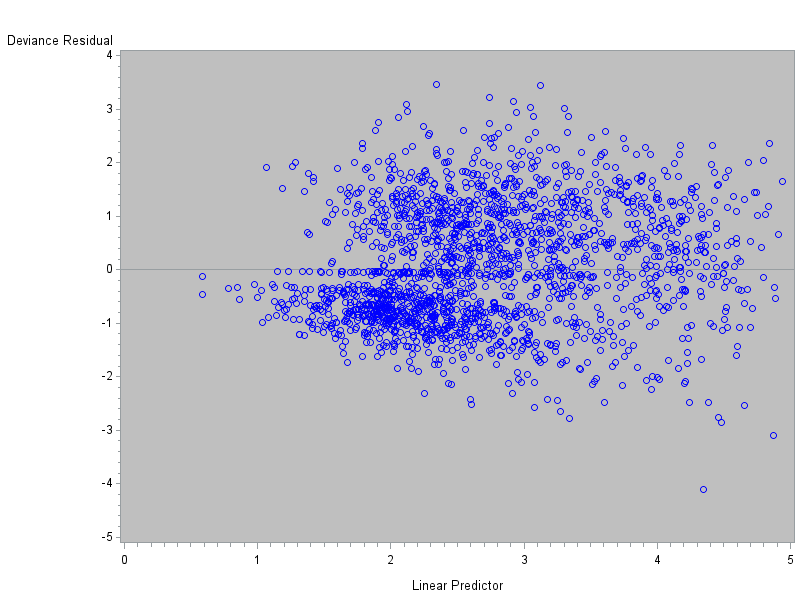 |
| --- | --- |
| C  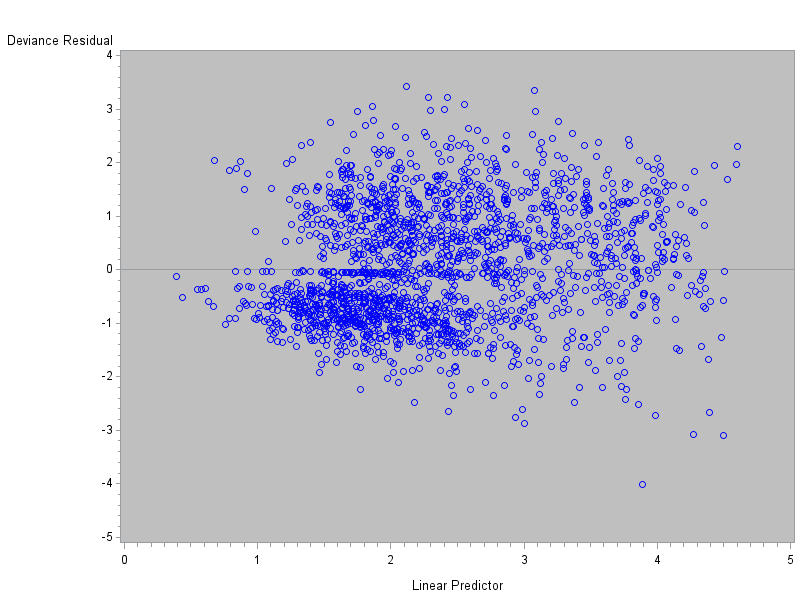 | D  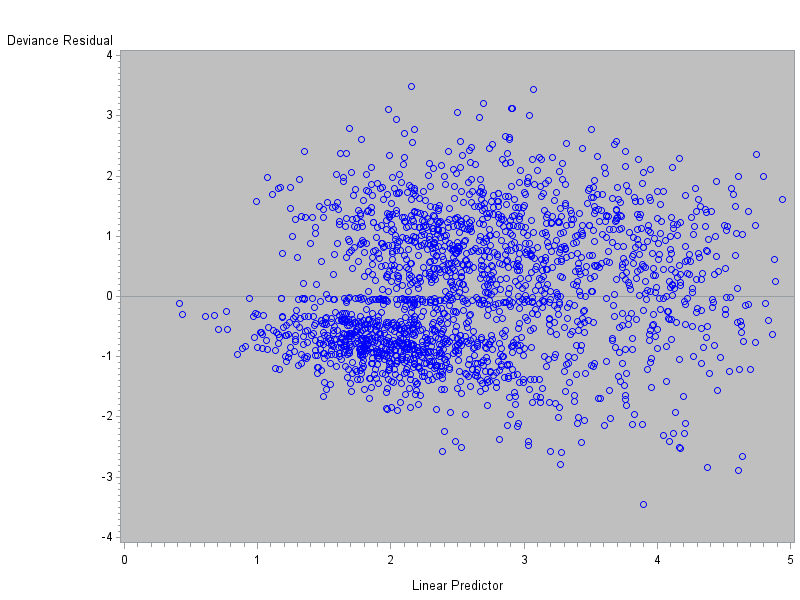 |

**Figure E3. Deviance plots for Cox models in the main analyses.**
